# New Advances in Improving Bone Health Based on Specific Gut Microbiota

**DOI:** 10.3389/fcimb.2022.821429

**Published:** 2022-07-04

**Authors:** Qihui Yan, Liping Cai, Weiying Guo

**Affiliations:** Department of Endocrinology and Metabolism, The First Hospital of Jilin University, Changchun, China

**Keywords:** gut microbiota, specific bacteria, treatment, bone health, osteoporosis

## Abstract

The gut microbiota has been shown to play an important role in the pathogenesis of various diseases, including metabolic diseases, cardiovascular diseases, and cancer. Recent studies suggest that the gut microbiota is also closely associated with bone metabolism. However, given the high diversity of the gut microbiota, the effects of different taxa and compositions on bone are poorly understood. Previous studies demonstrated that the mechanisms underlying the effects of the gut microbiota on bone mainly include its modulation of nutrient absorption, intestinal permeability, metabolites (such as short-chain amino acids), immune responses, and hormones or neurotransmitters (such as 5-hydroxytryptamine). Several studies found that external interventions, such as dietary changes, improved bone health and altered the composition of the gut microbiota. This review summarises the beneficial gut bacteria and explores how dietary, natural, and physical factors alter the diversity and composition of the gut microbiota to improve bone health, thereby providing potential new insight into the prevention of osteoporosis.

## Introduction

The human gut is home to ten trillion diverse symbionts consisting of several types of gut microbiota, including bacteria, fungi, archaea, viruses, and parasites. Recently, the role of intestinal microbiota in various diseases has become a research topic of high interest. Sev eral studies have shown that gut microbes are involved in the pathogenesis of metabolic diseases, such as diabetes (Adeshirlarijaney and Gewirtz, 2020), obesity (Miyamoto et al., 2019), and non-alcoholic fatty liver disease (Aron-Wisnewsky et al., 2020; Fan and Pedersen, 2021), and are closely associated with cardiovascular diseases (Kazemian et al., 2020), autoimmune diseases (Zhang et al., 2020), and cancer (Garrett, 2019). In recent years, intestinal microbiota have been suggested to play an important role in regulating bone metabolism, leading to the emergence of a new interdisciplinary research field termed “osteomicrobiology”, which bridges the gap between bone physiology, gastroenterology, immunology, and microbiology (Ohlsson and Sjögren, 2018).

Bone is a vital organ composed of collagen and calcium phosphate. It provides physical support and stores minerals, such as calcium and phosphorus, for the body. Its physiological function is maintained through the dynamic process of continuous bone remodeling (balance between osteoblasts and osteoclasts), which is strictly regulated by the organism. Dysregulation of this process leads to abnormal bone remodeling in postmenopausal women and secondary osteoporosis (Kim et al., 2020). Osteoblasts, the main cells that regulate bone formation, secrete extracellular matrix proteins. The expression of Runt-related transcription factor 2 (RUNX2) is a marker of osteoblast maturation. Osteoclasts originate from the hematopoietic lineage and differentiate into mature osteoclasts to function in bone resorption *via* macrophage colony-stimulating factor and nuclear factor-κB receptor-activating factor pathways. Mesenchymal stem cells (MSCs) are a pluripotent, non-hematopoietic stem cell population first identified in the bone marrow with the ability to differentiate into mature cell types of mesenchymal tissue (Salhotra et al., 2020). Both osteoblasts and osteoclasts can be differentiated from bone marrow MSCs.

There is growing evidence have shown a strong association between gut microbes and bone (Chen et al., 2017b; Lu et al., 2021a). As a focus of current research, gut microbes are a new target for improving bone mass by regulating bone metabolism and influencing dynamic bone homeostasis (Ibáñez et al., 2019; Behera et al., 2020; Cooney et al., 2020). The mechanisms mainly include their regulation of nutrient absorption (Weaver, 2015), intestinal permeability, metabolites [such as short-chain amino acids (SCFAs)] (Zaiss et al., 2019), immune responses (D'Amelio and Sassi, 2018; Lorenzo, 2021), and hormones (Yan et al., 2016) or neurotransmitters (such as 5-hydroxytryptamine) (Ding et al., 2020). Exploring the relationship between gut microbes and bone will provide new insights for clinical and provide a foundation for the prevention and treatment of bone diseases.

Importantly, both animal and clinical studies have found that bone mass is associated with the diversity and composition of the gut microbiota (Das et al., 2019; Rios-Arce et al., 2020; Tyagi et al., 2021). This review summarises the recent studies on the effects of gut microbes on bone. It also describes the relationship between the taxonomic characteristics of gut microbes and bone health and explores how various interventions affect bone quality by regulating the diversity, composition, and metabolites of gut microbes. This review explores new approaches for improving bone mass and provides novel strategies for epidemiological, basic, and clinical research.

## Intestinal Microbiota Classification Characteristics and Bone Health

Increasing evidence indicates that the diversity and composition of the gut microbiota play an important role in bone health and other processes. For some animal experiments, Schepper et al. (2019) demonstrated that after the application of broad-spectrum antibiotics, re-propagation of the intestinal flora for 4 weeks led to gut microbiota dysbiosis, alterations in intestinal permeability, and reduced bone density in the femoral trabeculae of mice. A rat study concluded that compared with the sham surgery group, osteoclast activity was significantly enhanced, and osteoblast activity was reduced in the Roux-en-Y gastric bypass surgery group, resulting in serious bone loss and defective autophagy. Therefore, after bariatric surgery, alterations in the gut microbiota may contribute to impaired bone mass (Shang et al., 2021). The same conclusion was reached in another animal study (Ma et al., 2020a; Cheng et al., 2021). In clinical studies, Xu et al. (2020) analysed the composition of intestinal microbiota by 16S rDNA amplification and sequencing and found significant differences in gut microbial diversity and composition between patients in the primary osteoporosis group and healthy controls. He et al. (2020) measured the faecal microbiota of 106 postmenopausal women with osteopenia, osteoporosis, and normal bone mineral density and found that bacterial richness and diversity were decreased in postmenopausal women with osteoporosis. A large study based on a Chinese population provided robust evidence that osteoporosis was associated with beta diversity, and composition of the gut microbiota (Ling et al., 2021). A study based on 16S rRNA sequencing came to the same conclusion that the diversity of the gut microbiota distinguished patients with osteoporosis and osteopenia from normal controls, with Gemmatimonadetes and Chloroflexi higher at the phylum level in the osteoporosis and osteopenia groups than in control group (Wang et al., 2017).

The above animal experiments and clinical studies point to a strong correlation between reduced diversity and altered composition of gut microbes and changes in bone mass and the development of bone disease. However, the change in the composition of gut microbiota was inconsistent throughout the studies observed so far, and often with opposite directions of the change (Knudsen et al., 2021). As the most abundant and diverse microbiota in the human body, the intestinal microbes are mainly composed of seven bacterial phyla, including Firmicutes, Bacteroides, Actinobacteria, Proteobacteria, Fusobacteria, Verrucomicrobia, and Cyanobacteria, and the first four phyla account for over 90% of the gut microbiota (Adak and Khan, 2019). Considering the high taxonomic diversity of gut microbes, the effects of specific gut microbes on bone health are important to investigate in terms of phylum, order, family, and genus.

### Firmicutes

Several previous studies found that bacterial abundance at the orders, families, and genera level belonging to Firmicutes showed an incoherent relationship with bone mass. Clostridiales showed a negative correlation with bone health. A study explored the correlation between gut microbiome signature and BMD alterations in the elderly Chinese population and found a positive correlation between the abundance of Firmicutes and Clostridiales and BMD in postmenopausal women, and suggested that Firmicutes and Clostridiales may play an important role in maintaining normal skeletal physiological conditions (Wang et al., 2022). Other studies have suggested the opposite, suggesting that Clostridiales showed a negative association with bone health. In a study related to Clostridiales, MA et al. performed 16S rRNA and metagenome sequencing using forty female rats randomly divided into ovariectomy or control groups. The experiment found that there was a high abundance of Clostridium in the ovariectomy group and that Ruminococcus belonging to the same order might be the pathogenic bacteria of steroid deficiency-induced osteoporosis (Ma et al., 2020b). The same conclusion was reported in a clinical study by NI et al. They identified specific bacteria taxa that regulated bone mass variations *via* a Mendelian randomization approach and found that Clostridiales and Lachnospiraceae were negatively associated with heel bone density (Ni et al., 2021). In contrast, a clinical study in China by LI et al. found a reduced abundance of Lachnospiraceae in individuals with low bone density and a positive correlation between BMD, T-scores, and Lachnospiraceae by high-throughput 16S rRNA gene sequencing of 102 faecal samples (Li et al., 2019a). The contradictory conclusions indicate that bacteria in the same family may produce different effects, and differences exist in the gut microbial composition between races and regions.

Similarly, contradictory conclusions were reported for the genus Clostridium belonging to Clostridiaceae. Tyagi et al. (2021) found that segmented filamentous bacteria belonging to the genus Clostridium induced the expansion of Th17 cells in the intestine, negatively affecting bone health by transferring faecal microorganisms from mouse mothers to their offspring. In contrast, numerous studies have found that butyrate-producing bacteria positively affect bone health. Clostridium butyricum belonging to the genus Clostridium has demonstrated potential protective or ameliorative effects in several human diseases, including intestinal diseases, neurodegenerative diseases, and metabolic diseases et al. (Stoeva et al., 2021). Studies have shown that Clostridium butyricum can alleviate the imbalance of gut flora by promoting the growth of probiotics and can maintain the intestinal epithelial barrier (Hagihara et al., 2018; Li et al., 2018). Recently, studies have further demonstrated the beneficial effects of Clostridium butyricum on bone-related diseases. A study in rats demonstrated that Clostridium butyricum alleviated bone loss induced by alterations in gut microbiota by promoting osteoblast autophagy after bariatric surgery (Shang et al., 2021). Another Chinese clinical study demonstrated a positive correlation between Roseburia (primarily produce butyric acid) and BMD and T-scores (Li et al., 2019a). Although studies have indicated that Clostridialesis not well identified in the regulation of bone health, individual taxa within the same order show a positive effect. The specific bacteria that dominate these effects on bone are not fully known.

Regarding Lactobacillus, there are also contradictory conclusions. Wen et al. (2020) observed a significant increase in bacterial abundance at the Lactobacillus order, family, and genera levels in a mouse model of postmenopausal osteoporosis. A clinical trial measured the faecal microbiota of 108 older adults and showed an increased abundance of the genus Lactobacillus in the group of patients with osteoporosis. After excluding differences related to BMI and medications, the study suggested an adverse effect of Lactobacillus on osteoporosis (Das et al., 2019). In contrast, part of Lactobacillus belonging to this genera, such as Lactobacillus acidophilus, Lacticaseibacillus paracasei GMNL-653, and Lactobacillus reuteri et al, were thought to positively regulate bone health (Dar et al., 2018; Lee et al., 2021; Jhong et al., 2022). Britton et al. showed that Lactobacillus reuteri suppressed ovariectomy-induced increases in bone marrow CD4+ T lymphocytes and inhibited estrogen deficiency-induced bone resorption and bone loss (Britton et al., 2014).A study using a mouse model with gut microbial dysbiosis after antibiotic treatment found that Lactobacillus reuteri prevented osteoblastopenia and increased osteoclast activity, thereby preventing trabecular bone loss in the femur and spine and exerting a positive skeletal effect. In addition, a similar clinical trial concluded that Lactobacillus reuteri reduced further bone loss in elderly women with pre-existing low bone density (Nilsson et al., 2018). These studies provide further evidence that even bacteria within the same genus have significantly different effects on bone. This helps us better understand the relationship between different bacteria and bone, thereby indicating therapeutic targets for future research (Schepper et al., 2019).

### Bacteroidetes

Several previous clinical studies have pointed to a higher abundance of Bacteroides in patients with osteoporosis. In China, He et al. (2020) analysed the faecal microbiota profiles of 106 postmenopausal women with osteopenia, osteoporosis, or normal BMD and demonstrated decreased bacterial richness and diversity and higher abundances of Bacteroides and Parabacteroides in postmenopausal osteoporosis. These findings indicated a negative effect of bacteria in these two genera on bone health. Likewise, a study found that Bacteroides was associated with reduced bone mineral density and T-value at the lumbar spine, with a link to the risk of osteoporosis by measuring the abundance of gut microbiota in patients with osteoporosis (Wei et al., 2021). In contrast, a study of postmenopausal women in Japan concluded that the fracture incidence was significantly higher in the low Bacteroides group, with a 5.6-times higher risk ratio of fracture history. The study suggested a positive effect of the genus Bacteroides on bone metabolism and fracture incidence, but there was no significant difference in bone mineral density (BMD). In addition, Rikenellaceae was more abundant in the low BMD group and the high tartrate-resistant acid phosphatase 5b group, which may have a negative effect on bone resorption and bone mineral density (Ozaki et al., 2021). Opposite findings were also found for the effect of Prevotellaceae on bone. Prevotellaceae are considered pro-inflammatory bacteria and are also associated with the synthesis of SCFAs. Previous findings indicate that Prevotella may be a clinically important pathobiont for steroid deficiency-induced osteoporosis. However, some studies have reported that the abundance of Prevotella is more than three times higher in normal populations than in patients with osteoporosis. Prevotella may prevent estrogen deficiency-induced bone loss *via* the intestinal bone axis and is thought to be a therapeutic agent and target for osteoporosis (Wang et al., 2017; Wang et al., 2021). Interestingly, an animal study reported that Prevotella increased rapidly and maintained high abundance in the early and middle stages of osteoporosis but gradually decreased in the later stages (Ma et al., 2020b). Further research on the effects of this genus on bone is needed. Similar to Firmicutes, there is considerable variation in the skeletal effects of the bacteria in the Bacteroidetes phylum, which still needs to be explored at the subgeneric level.

### Firmicutes/Bacteroidetes Ratio

Most studies have shown that a high Firmicutes/Bacteroidetes ratio (F/B ratio) is a microbiological feature of several diseases, such as diabetes, obesity, and hypertension (Magne et al., 2020; Zou et al., 2020). Similarly, researchers have focused on the relationship between osteoporosis and the F/B ratio, which has not yet been conclusively established. MCCABE et al. indicated that exposure to a high-fat diet induced changes in microbial composition and reduced the volume of trabeculae in the tibia and vertebrae. They also found that exercise altered the compositions of gut microbiota *via* reducing the F/B ratio. This ratio was negatively correlated with bone volume, and this experiment also demonstrated a negative effect of Clostridium and Lachnospiraceae on bone (McCabe et al., 2019). An increase in the F/B ratio was observed after ovariectomy and a high-fat diet in other studies. The F/B ratio may be a valid biomarker in steroid-deficient osteoporosis (Ma et al., 2020b). Another study also observed a significant increase of the F/B ratio in a mouse model of postmenopausal osteoporosis and suggested that an imbalance of gut microbiota may lead to overactivation of self-immunity which is accepted osteoporosis pathogenesis (Wen et al., 2020; Lu et al., 2021b). In contrast, an animal study in China showed a significantly lower diversity of gut microbiota and lower F/B ratios in 22-month-old rats with osteoporosis by applying 16SrRNA metagenomic sequencing (Ma et al., 2020a). Different ethnic differences may lead to contradictory results. Therefore, the F/B ratio as a significant indicator of osteoporosis remains to be further explored.

### Other Taxa

Recent studies have shown that the abundance of Proteobacteria reflected instability and metabolic disturbances in the intestinal microbial community (Litvak et al., 2017). Both quantitatively and functionally, MA et al. demonstrated using animal experiments that Helicobacter pylori belonging to Proteobacteria might be a potential pathogenic factor in senile osteoporosis (Ma et al., 2020a). He et al. (2020) also observed a high abundance of some bacteria belonging to Proteobacteria in postmenopausal osteomalacia, such as Klebsiella, Escherichia coli, Enterobacter, Citrobacter, and Pseudomonas.

Several previous studies have reported that Actinobacteria exhibits a positive effect on bone. Bifidobacteriaceae are intestinal bacteria that reduce intestinal inflammation and could be considered as a new potential target to prevent osteoporosis (Parvaneh et al., 2015; Wallimann et al., 2021). A study showed that a decreased number of Actinobacteria (especially Bifidobacteriaceae) was associated with an enhancement of gut permeability, leading to the translocation of lipopolysaccharide into the serum. The change of lipopolysaccharide contributed to bone mass *via* inflammation-relevant pathways (Duca et al., 2013). A study based Chinese elderly population inferred that the genetic variants of the LGR6 gene may increase the osteoporosis risk by reducing the abundance of Actinobacteria, Bifidobacteriaceae, and Bifidobacterium (Di et al., 2021). The study alsoconcluded that Actinobacteria (including Bifidobacteriaceae) were positively correlated with BMD (McCabe et al., 2019).

In conclusion, Actinobacteria, Roseburia, butyrate-producing Clostridia, Lactobacillus acidophilus and Lactobacillus reuteri are beneficial intestinal bacteria that might promote bone health, whereas Proteobacteria, Rikenellaceae, Ruminococcaceae, segmented filamentous bacteria, and Parabacteroides exert an opposite skeletal effect. Contradictory findings regarding the effects of the Firmicutes/Bacteroidetes ratio, Lachnospiraceae, Bacteroides, and Prevotella on bone warrant further investigation. Clinical and animal studies have revealed different effects of various taxa on bone, thereby guiding us to further investigate the effects of specific bacteria at the genus level on bone mass. Thus, for the improvement of bone health, focusing on new therapeutic approaches targeting specific bacteria in the gut microbiota and their potential roles in increasing bone strength or preventing fractures is necessary.

## Interventions With Specific Gut Microbiota to Improve Bone Health

The therapeutic use of gut microbes as a target for skeletal diseases has become a current hot topic. Gut microbial dysbiosis is one of the important pathogenic mechanisms underlying primary osteoporosis, and several therapies based on gut microbes are used to alleviate primary osteoporosis (Ohlsson and Sjögren, 2015), such as antibiotics (Cox et al., 2014), probiotics (Ohlsson et al., 2021), prebiotics (oligosaccharides and polysaccharides) (Porwal et al., 2020; Zhang et al., 2021), and faecal bacteria transplants (Ding et al., 2020). From the perspective of the gut microbes, which are beneficial to bone health, some studies have found that external factors, as a novel approach, could intervene in the composition of the gut microbes to increase beneficial bacteria and reduce the abundance of bacteria that are detrimental to bone health, and subsequently influence bone metabolism and bone mass changes. Non-pharmaceutical preparations for the treatment of osteoporosis are becoming popular. However, there are limited studies and data available in this field. This review aims to summarise how other novel interventions alter gut microbial diversity, composition, and metabolites to subsequently regulate dynamic bone homeostasis, influence bone mass, and improve bone health. In addition, this article provides potential options and new directions for targeting specific gut microbes to prevent osteoporosis by collecting literature from recent years. The following focuses on the evidence for an association between intervention factors, the changes in gut microbiota, and bone health (**Table 1**).

**Table 1:** Ingredients that contribute to bone health reported in previous studies.

Intervenes	Active ingredients	Research design	Changes in gut miacrobiota	References
**Montgomery**	anthocyanins	*In vivo*, preclinical mouse model of gonadectomy	↑ Ruminococcus 1 ↑ Prevotellaceae UCG-001 ↑ Coriobacteriales	Sato et al. (2021)
**Tuna bone powder**	Calcium and phosphate	*In vivo*, glucocorticoid-induced osteoporosis in female mice	↑ Firmicutes, Faecalibacterium ↑ Parabacteroide ↓ Helicobacter ↓ Prevotella ↓ Proteobacteria	Li et al. (2020b)
**Kefir-fermented peptides**	Bioactive peptides	*In vivo*, Ovariectomy (OVX)-induced osteoporosis mouse model	↑ Firmicutes/Bacteroidetes ratio ↑ Alloprevotella ↑ Anaerostipes, Ruminococcus 1, Streptococcus ↑ Parasutterella	Tu et al. (2020)
**Duck egg white-derived peptide Val-Ser-Glu-Glu**	Bioactive peptides	*In vivo*, *in vitro*, OVX-induced osteoporosis rat model	↑ Bacteroides ↓ Veillonellaceae, Ruminococcus, Roseburia ↓ Prevotellaceae ↓ Fusobacterium	Guo et al. (2019)
**Tilapia nilotica fish head lipids (THLs)**	DHA	*In vivo*, OVX-induced osteoporosis rat model	↑ Oscillospira Roseburia Dubosiella ↓ Alistipes	Zhu et al. (2021)
**Puerarin**	7,4′-dihydroxy-8-C-glucosylisoflavone	*In vivo*, *in vitro*, OVX-induced osteoporosis rat model	↑ Bacteroidia, Bacteroidales_S24−7_group, Prevotellaceae, Alloprevotella ↑ Lactobacillus ↑ Bifidobacterium ↓ Firmicutes/Bacteroidetes ratio ↓ Clostridia, Ruminococcaceae, Faecalibacterium, Lachnospiraceae, Erysipelatoclostridium ↓ Melainabacteria ↓ Desulfovibrionaceae, Desulfovibrio	Li et al. (2020a)
**Arecanut seed polyphenol**	Polyphenol	*In vivo*, OVX-induced osteoporosis rat model	↑ Fimicutes ↑ Proteobacteria ↓ Alistipes	Mei et al. (2021)
**Green tea lyphenols and annatto-extracted tocotrienols**	Polyphenol and vitamin E	*In vivo*, mouse model of high-fat diet-induced obesity	↑ Clostridium (in the Clostridiaceae family), Subdoligrannulum variabile, Clostridum saccharogumia ↑ Akkermansia muciniphila ↓ Ruminococcus, Dorea longicatena, Clostridium (in the Lachnospiraceae family), Clostridium symbiosum	Elmassry et al. (2020)
**Agastache rugosa ethanol extract**	Acacetin (Aca) and tilianin (Til, 7-O-β-glucoside)	*In vivo*, *in vitro*, OVX-induced osteoporosis mouse model	↑ Lactobacillus ↓ Clostridium	Hong et al. (2021)
**Fructus Ligustri Lucidi**	Flavonoids, terpenoids, phenylethanoid glycosides, phospholipids, polysaccharides	*In vivo*, mouse model of age-related osteoporosis	↑ Actinobacteria, Bifidobacterium ↑ Erysipelotrichi, Coriobacteriia ↑Lactobacillus ↓ Sutterella, Desulfovibrio ↓ Coprococcus	Li et al. (2019b)
**Geranylgeraniol (GGOH)**	geranylgeraniol	*In vivo*, mouse model of high-fat diet-induced obesity	↑ Butyricicoccus pullicaecorum ↓ Dorea longicatena	Chung et al. (2021)
**Erythrina cortex extract**	isoflavonoids	*In vivo*, OVX-induced osteoporosis mouse model	↑ Ruminiclostridium ↓ Alistipes Akkermansia	Xiao et al. (2021)
**Cinnamic acid**	cinnamic acid	*In vivo*, OVX-induced osteoporosis mouse model	↑ Lactobacillus ↓ Desulfovibrionaceae	Hong et al. (2022)
**Infrared light exposure**	Infrared light	*In vivo*, OVX-induced osteoporosis rat model	↑ Clostridiaceae * *1, Erysipelotrichaceae, Lactobacteriaceae, Peptostreptococcaceae ↑ Porphyromonadaceae, Prevotellaceae ↓ Saccharibacteria ↓ Firmicutes/Bacteroidetes ratio ↓ Desulforibrionaceae	Lu et al. (2021)
**Ultraviolet light supplementation**	ultraviolet light	*In vivo*, OVX-induced osteoporosis rat model	↑ Firmicutes ↓ Proteobacteria	Cui et al. (2021)
**Warmth exposure**	34°C	*In vivo*, OVX-induced osteoporosis in 16-week-old mice	↑ Akkermansia ↑ Clostridium_sensus_stricto_1 ↑ Parabacteroides ↑ Turicibacter ↑ Ruminiclostridium_6 ↑ Rhodospirillales ↓ Butyricicoccus, Peptococcaceae	Chevalier et al. (2020)

↑ means an increase in gut microbiota. ↓ means an decrease in gut microbiota.

### Diet

There may be a relationship between anthocyanins and the composition of the gut microbiota, including increased overall quantity and specific microbial growth. anthocyanins have beneficial effects on chronic diseases prevalent in the elderly (Lee et al., 2018; Hair et al., 2021). Several studies have shown that blueberries, which are rich in anthocyanins, have been shown to prevent bone loss (Zhang et al., 2011; Domazetovic et al., 2020). Whether blueberries can influence bone health by affecting the diversity and composition of gut microbes. Sato et al. (2021) used a preclinical model of gonadectomy in mice to simulate human sex hormone deficiency to verify this hypothesis. They added a blueberry cultivar (Montgomery) to the diet and found that Montgomery increased the diversity of the gut microbiota in ovariectomized female mice and enhanced the endogenous antioxidant response, thereby protecting the mice from musculoskeletal loss and body weight changes induced by ovariectomy. However, androgen-induced bone loss in males was not protected. The mechanism involves bypassing nuclear factor E2-related factor 2, which is a classic antioxidant transcription factor, and canonical estrogen receptor signaling.

Tuna bone powder (TBP) is mainly composed of calcium and phosphate and has a stronger promoting effect on bone metabolism than calcium carbonate. To investigate whether TBP regulates the microbial composition of the gut. Li et al. (2020b) found that the abundance of the anti-inflammatory bacterium Firmicutes was increased, the pro-inflammatory bacteria Proteobacteria and Prevotella were decreased, and the content of short-chain fatty acids was increased in the TBP-treated group. This study demonstrated that TBP promoted osteoblastogenesis, inhibited osteoclastogenesis, suppressed pro-inflammatory cytokine release, repaired intestinal epithelial barrier dysfunction, and prevented systemic inflammation *via* co-regulating nuclear factor-kappa B and Wnt/β-catenin signaling pathways. Subsequently, these changes alleviated osteoporosis.

Currently, some bioactive peptides have been identified and shown to be effective in preventing bone loss in ovariectomized rats, promoting bone metabolism (Reddi et al., 2018; Chen et al., 2022). Bioactive peptides also have a regulatory effect on intestinal microbes (Ashaolu, 2020). Kefir-fermented peptides (KPs) are bioactive peptides produced through the degradation of proteins in dairy milk by the probiotic microflora in Kefir grains. A few peptides (mainly from casein) have hypotensive, anti-inflammatory, and immunomodulatory effects. Kefir has been shown to improve bone mass and microarchitecture in an ovariectomised rat model of postmenopausal osteoporosis (Chen et al., 2015). Regarding its relationship with gut microbiota, Tu et al. (2020) designed a related study in which an ovariectomised mouse model was used to simulate oestrogen-related bone loss. Following the feeding of KPs to rats for 8 weeks, microbiota richness and diversity were increased in the KPs group, the abundance of Alloprevotella, Anaerostipes, and Ruminococcus 1 was restored to levels similar to those in the sham-operated group, and the levels of serum alkaline phosphatase and C-telopeptide of type I collagen were decreased. Moreover, higher bone mineral density, trabecular bone number, and bone volume were observed in the KPs group compared with the sham-operated group.

Guo et al. (2019) previously showed that the duck egg white-derived peptide Val-Ser-Glu-Glu (VSEE), as a bioactive peptides, promoted calcium absorption by activating calcium channels and indicated that it might be a co-factor in the prevention of osteoporosis. Guo et al. (2019) investigated the effects of VSEE on anti-osteoporotic activity and intestinal microbiomes in ovariectomised rats, revealing significantly decreased Veillonellaceae, Prevotellaceae, Fusobacterium (Bacteroidaceae 1), Ruminococcus, and Roseburia and increased Bacteroides in the VSEE group. In addition, the proliferation, differentiation, and mineralization of preosteoblasts were enhanced. VSEE played an important role in protecting against bone loss in ovariectomised rats. The relevant mechanisms were attributed to the activation of the Wnt signaling pathway and the regulation of the expression of Runx2, osteoprotegerin, and other bone formation genes through inducing transient increases in calcium.

In a study using an ovariectomized osteoporosis rat model, Tilapia nilotica fish head lipids (THLs) significantly reduced the levels of Alistipes in the gut, while increasing the abundance of beneficial bacteria, and suggested that THLs may be a functional factor with antiosteoporotic activity. THLs are also rich in docosahexaenoic acid, which has been shown to regulate the intestinal microbiota and promote bone formation (Che et al., 2021; Zhu et al., 2021).

The phenolic compounds in prunes may alter the composition of gut microbes, and in addition, prunes have a protective effect on bone health, which is considered to be associated with anti-inflammatory and antioxidant pathways (Duda-Chodak et al., 2015; Arjmandi et al., 2017). In addition to prunes, other polyphenol-rich foods exhibit similar biological activity in the prevention of bone loss (Lucas et al., 2020). Future research should aim to investigate whether this protective effect is associated with alterations in gut microbes (Damani et al., 2022).

Foods rich in anthocyanins, calcium, unsaturated fatty acids, bioactive peptides, and polyphenols regulate the diversity and composition of the intestinal microbiota to a certain extent and thus have a beneficial effect on the bone. This idea should be supported by a large number of experiments and data, especially clinical trials. In addition, researchers should pay more attention to the active ingredients in foods that are beneficial for bone health. This suggests that people at risk of osteoporosis in the future can take care of their diet in several ways. In addition to a regular calcium-rich diet, other safe foods that are good for bone health can be consumed to prevent osteoporosis.

### Natural Substances

Several studies indicate that puerarin may be used to prevent osteoporosis (Liu et al., 2016; Xiao et al., 2020). In addition, the anti-osteoporosis effect of puerarin was found to be associated with modulations in gut microbiota in an animal study. Li et al. (2020a) established an osteoporosis model in ovariectomized rats to investigate the mechanism of the gut/bone axis. Puerarin treatment increased bone mineral density and improved the bone micro-environment *via* regulating the composition of gut microbiota, thereby enhancing intestinal mucosal integrity and reducing systemic inflammatory responses. After puerarin intervention, the gut microbial diversity, abundance of Lactobacillus, Bacteroidales, Prevotellaceae, and Bifidobacterium, and content of SCFAs were increased, whereas Lachnospiraceae and Ruminococcaceae belonging to Clostridiales and the F/B ratio were decreased.

Polyphenols are a natural active substance extracted from plants with reported health-promoting effects, including immunoregulation, anti-obesity, microbiota modulation, anti-inflammatory, and anti-osteoporotic activities (Wong et al., 2020; Wan et al., 2021). An experimental animal study was conducted to investigate the association between arecanut seed polyphenol (ACP) and anti-osteoporosis activity. An ovariectomy rat model of osteoporosis was used, and rats were randomly divided into four groups: the ovariectomy group, E2 treatment group, high-dose ACP group, and low-dose ACP group. The study showed a decreased abundance of Alistipes, which belongs to the Rikenellaceae family, with an inhibitory effect on bone metabolism. In addition, the abundance of Firmicutes and Proteobacteria was markedly increased in the ACP treatment group. This change contributed to increased lysozyme expression by maintaining the number of estrogen-deficient Panthera cells, which improved osteoporosis *via* regulating the immune system, enhancing the microstructure of trabeculae, and increasing the number, thickness, and spacing of trabeculae (Mei et al., 2021). Another animal experiment concluded that green tea polyphenols promoted bone health in obese mice *via* regulating the gut microbiota (Elmassry et al., 2020). In a high-fat diet-induced obesity mouse model, Akkermansia muciniphila (Proteobacteria) and Clostridium saccharogumia belonging to the beneficial Clostridium genus were increased, and Firmicutes were decreased in the annatto-extracted tocotrienols combined with green tea polyphenols supplemented group. The osteoprotective effect was associated with the promotion of vitamin K synthesis.

Patchouli has various bioactivities, including antioxidant, anti-fungal, anti-inflammatory, and anti-obesity. Previous studies reported that Agastache rugosa extracts have an inhibitory effect on osteoblast differentiation (Jang et al., 2020). Hong et al. (2021) used an ovariectomy mouse model and found that an ethanol extract of Agastache rugosa (EEAR) and 7-O-β-glucoside reversed the ovariectomy-induced changes in intestinal microbiota, such as increasing Lactobacillus and decreasing Clostridium. This extract had a therapeutic effect on postmenopausal osteoporosis by promoting osteogenic differentiation through bone morphogenetic protein, transforming growth factor β, and Wnt signaling pathways. EEAR may be a potential candidate for improving postmenopausal osteoporosis.

Fructus Ligustri Lucidi (FLL) is believed to have antioxidant, anti-ageing, and osteoprotective functions and is used to prevent and treat osteoporosis (Chen et al., 2017a; Liu et al., 2021). Whether this effect is related to the diversity and composition of the gut microbiota was investigated in an animal study. Ageing-related osteoporosis in mice was induced by constant exposure to D-galactose/sodium nitrite for 90 days, and mice were randomly divided into normal control, ageing, ageing+VE, and ageing+ FLL groups. Interestingly, the study concluded that in the FLL treatment group, the F/B ratio was comparable to that in the normal control group, whereas the abundance of Actinobacteria was significantly higher than in the other groups, with Coriobacteriia being its critical bacterial taxon. FLL ameliorated the reduced numbers of Lactobacillus and Bifidobacterium following FLL intervention. This study suggests that the mechanisms protecting against ageing-related bone mass may be linked to the diversity of gut microbiota, inhibition of antioxidant activity, and levels of monooxygenase-3 and trimethylamine-N-oxide (Li et al., 2019b).

Previous studies have suggested that geranylgeraniol (GGOH) supplementation would benefit bone health, probably in part through modulating the mevalonate pathway. The study by Chung et al. demonstrated that this effect may be attributed to the alteration of gut microbes by GGOH (Chung et al., 2021). Some studies have noted that the antiosteoporotic effects of isoflavonoids are mediated through modulation of bone turnover and improvement of bone microarchitecture (Zhang et al., 2010; Tousen et al., 2016; Lambert et al., 2017). Similarly, a study by Xiao et al. indicated that the skeletal protective effect of isoflavonoids-rich extract of Erythrina cortex was associated with alterations in several specific gut bacteria (restoration of Ruminiclostridium and inhibition of Alistipes and Akkermansia) and an increase in SCFAs (Xiao et al., 2021). Cinnamic acid is a natural compound found in dietary plants and has a variety of biological properties. Cinnamic acid was first reported to increase the diversity of intestinal microbes and modulate specific intestinal microbes such as Lactobacillus and Desulfovibrionaceae to induct osteoblast differentiation and suppress bone loss by Hong et al. (Hong et al., 2022).

A variety of natural substances from plant extracts or a particular plant such as puerarin, polyphenols, extract of Agastache rugosa, FLL, isoflavones, and cinnamic acid have a skeletal-promoting effect, which is associated with an altered gut microbial composition. This suggests that researchers should focus on this aspect and explore potential mechanisms for the effects of active ingredients on bone. More clinical trials should be designed in the future to promote non-pharmacological interventions as therapeutic measures.

### Physical Factors

Prolonged exposure to artificial lighting has a negative impact on bone health, leading to bone loss and osteoporosis. Infrared and ultraviolet light supplementation is used to simulate sunlight and regulate bone metabolism. To clarify whether this effect is related to gut microbes. Lu et al. (2021) supplemented white LED irradiation with infrared light in rats for three consecutive months and found that the abundance of Saccharibacteria and F/B ratio were significantly reduced, whereas the amounts of Clostridiaceae 1, Erysipelotrichaceae, Peptostreptococcaceae, Porphyromonadaceae, and Prevotellaceae were increased. This study suggested that infrared supplementation increased BMD and positively affected bone metabolism in rats by influencing the gut microbiota, reducing inflammation, and increasing butyrate production. Experiments conducted by Cui et al. in osteoporotic rats similarly found that ultraviolet light could influence the diversity and composition of gut microbes, such as an increase of Firmicutes and a decrease of Proteobacteria, thereby regulating bone metabolism (Cui et al., 2021). This also demonstrated the positive effects of natural light exposure in the fight against osteoporosis. The current research opens up new ways to protect bone health for individuals who work under artificial lighting for long periods, so it is particularly important to create a “healthy light” as a long-term goal.

In another animal experiment, Chevalier et al. (2020) analysed the gut microbiota of 24-week-old female mice exposed to 34°C for 8 weeks and revealed increased intestinal microbial diversity in the warm-exposed group compared with the control group. In addition, the abundance of Ruminococcus 6, Akkermansia (Verrucomicrobiales), Clostridium 1, Rhodospirillales (Proteobacteria), and Parabacteroides was increased. Exposure to warm temperatures enhanced the biosynthesis of bacterial polyamines, particularly acetylated spermidine and spermine, thereby increasing trabecular bone volume and connective tissue density and thickness, affecting osteoblast activity, and decreasing osteoclast differentiation.

The above findings indicate the beneficial effects of gut microbiota on bone as a potential new target for prevention of osteoporosis based on dietary, natural, and physical factors. The main focus is promoting the growth and abundance of specific bacteria that are beneficial to the gut and bone. Research on exploring safe interventions to protect bone has some therapeutic value, and it is particularly important to explore specific intestinal and skeletal beneficial bacteria. Therefore, further research is necessary for new therapeutic interventions that can be used for long periods without side effects. Notably, all of the above studies were conducted in animals, and further clinical studies are necessary for the complex relationship between external factors, gut microbes, and bone health.

## Conclusion

The gut microbiota is closely linked to bone health, and its diversity and composition play an even more important role. Different taxa exhibit different skeletal effects, and it appears that Actinobacteria, Roseburia, butyrate-producing Clostridia, Lactobacillus acidophilus, and Lactobacillus reuteri are beneficial intestinal bacteria that might promote bone health. Dietary, natural, and physical factors have produced good results in animal studies and may be further applied in clinical experiments. The fact that the effects of diet and natural substances on bone concerning their active ingredients also provide new ideas for the development of active ingredients to improve osteoporosis. The aim of this review was to summarise the relationship between different taxa and bone health and describe the factors that influence bone quality by affecting the microbial composition of the gut, thereby providing potential candidates for the prevention and treatment of osteoporosis. Future research should be devoted to exploring the effects of different genera of bacteria on bone and identifying the dominant bacteria that are closely associated with bone health. Targeting specific gut-beneficial microbiota for intervention is necessary to develop novel therapeutic strategies for osteoporosis. Moreover, investigating the effects of various interventions on gut microbiota and bone in clinical settings is important.

## Author Contributions

QY designed and wrote this review. LC performed the paper selection. WG edited the manuscript. All authors contributed to the article and approved the submitted version.

## Funding

The study was supported by Natural Science Foundation of Jilin Province (No. 20200201306JC).

## Conflict of Interest

The authors declare that the research was conducted in the absence of any commercial or financial relationships that could be construed as a potential conflict of interest.

## Publisher’s Note

All claims expressed in this article are solely those of the authors and do not necessarily represent those of their affiliated organizations, or those of the publisher, the editors and the reviewers. Any product that may be evaluated in this article, or claim that may be made by its manufacturer, is not guaranteed or endorsed by the publisher.
